# Interaction between visual impairment and subjective cognitive complaints on physical activity impairment in U.S. older adults: NHANES 2005–2008

**DOI:** 10.1186/s12877-024-04739-2

**Published:** 2024-02-17

**Authors:** Jinyuan Chen, Haoyu Li, Biting Zhou, Xian Li, Yihua Zhu, Yihua Yao

**Affiliations:** 1 Department of Ophthalmology, the First Affiliated Hospital, Fujian Medical University, Fuzhou, China; 2 Department of Ophthalmology, National Regional Medical Center, Binhai Campus of the First Affiliated Hospital, Fujian Medical University, Fuzhou, China; 3 Clinical Research Center for Eye Diseases and Optometry of Fujian Medical University, Fuzhou, China; 4 Department of Ophthalmology, The Second Xiangya Hospital, Central South University, Changsha, P. R. China; 5 Hunan Clinical Research Centre of Ophthalmic Disease, Changsha, P. R. China; 6 Manchester Royal Eye Hospital, Manchester University NHS Foundation Trust, Manchester, UK; 7 Division of Pharmacy and Optometry, School of Health Sciences, Faculty of Biology, Medicine and Health, The University of Manchester, Manchester, UK

**Keywords:** NHANES, Visual impairment, Physical activity impairment, Subjective cognitive complaints, Interaction

## Abstract

**Background/aim:**

To investigate the independent relationships of visual impairment (VI) and Subjective cognitive complaints (SCC) with physical function impairment (PFI) and the interaction effect between VI and SCC on PFI in American older adults.

**Methods:**

The data of this cross-sectional study was obtained from the 2005–2008 National Health and Examination Survey (NHANES) conducted in the United States. The VI criterion included both subjective self-reported eyesight conditions and objective visual acuity test results. The self-reported questionnaires were utilized to determine PFI and SCC. According to the survey design of NHANS, original data were weighted to produce nationally representative estimates. Both the unweighted original data and weighted estimates underwent analysis. Crude and adjusted logistic models were employed to assess the pairwise associations among VI, SCC, and PFI. To assess the interactive effect, measures such as the relative excess risk due to interaction (RERI), attributable proportion due to interaction (AP), and synergy index (S) were calculated.

**Results:**

A total of 2,710 subjects (weighted *n* = 38,966,687) aged 60 years or older were included. Compared with subjects without subjective visual impairment (SVI), those with SVI had a significant positive association with PFI [weighted OR (95%CI): 3.11 (2.25, 4.31)]. After multi-variable adjusting, the relationship remained significant [weighted OR (95%CI): 1.90 (1.32, 2.72)]. Similarly, those with objective visual impairment (OVI) were positively associated with the risk of PFI in the crude model [weighted OR (95%CI): 2.35 (1.53, 3.61)] and adjusted model [weighted OR (95%CI): 1.84 (1.07, 3.17)]. Moreover, we found the association of SCC with an increased risk of FPI [crude weighted OR (95%CI): 5.02 (3.40, 7.40); adjusted weighted OR (95%CI): 3.29 (2.01, 5.38)]. Ultimately, the additive interaction showed there was a significant positive interaction term between SVI and SCC on PFI, while OVI and SCC did not.

**Conclusion:**

Both VI and SCC were significantly associated with PFI in elder adults. Besides, there was a significant synergistic interaction between SVI and SCC on PFI, which indicated the improvement of SVI and SCC may be beneficial for the prevention of PFI. For the elderly, especially those with multiple disabilities, comprehensive and targeted approaches are imperative to foster their overall well-being and health.

**Supplementary Information:**

The online version contains supplementary material available at 10.1186/s12877-024-04739-2.

## Introduction

Population ageing is an inevitable reality and an urgent social issue faced by many countries worldwide, its rate has continued to accelerate. The U.S. Centers for Disease Control and Prevention (CDC) analyzed, from 2011 to 2014, approximately 22.6 million (11.9%) working-age (18–64 years) U.S. citizens who had at least one disability, with nearly half of them experiencing one or more functional disabilities [1]. Thus, it is reasonable to speculate that more problems will become more pronounced in their old age. Furthermore, various studies reported that approximately 17–54% of community-dwelling older adults have one or more disabilities [2–4].

Among these disabilities, visual impairment (VI) is one of the prevalent causes of disability among the elderly. For individuals, VI not only interferes with daily activities and reduces the quality of life, but also increases the risk of comorbidities and medical expenses. In the year 2020, about 6.5 million adults aged 65 years and above were affected by VI, and this number is expected to increase two-fold by 2050 [5]. A previous study reported that glaucoma patients with greater levels of visual field loss exhibited decreased physical activities [6]. Besides, there were difficulties in walking and climbing steps for people with VI, especially as they age [7].

Additionally, cognitive decline is a prevalent condition with ageing [8]. Subjective cognitive complaints (SCC) are viewed as predictive indicators of objective cognitive decline and serve as a pre-clinical stage of Alzheimer’s disease [9]. Some evidence suggested that comparing the Parkinson’s disease patients without VI, individuals with Parkinson’s disease and VI performed worse in terms of cognitive competence and were more likely to progress to mild cognitive impairment [10].

Physical activity serves as an essential indicator of elderly health. The health benefits of physical activity for older adults have been extensively documented [11–13]. Moderate levels of physical activities have been shown to alleviate symptoms of anxiety and depression, improve brain function, protect against cognitive decline [14], and contribute to chronic disease management [15]. However, it is worth noting that the elderly with VI or SCC may not experience these benefits from physical activities. However, these associations remain to be further demonstrated in the population-based samples. Identifying the potential risk factors of physical functional impairment (PFI) in older adults and implementing targeted strategies are beneficial to improve their health and overall well-being.

Although numerous studies examined the associations between VI, SCC, and PFI, most previous studies only focused on two of these factors [16–18]. Rarely research on the integrated impacts of these disabilities, particularly among the elderly population. Therefore, this study aims to investigate the associations, specifically the interaction effects, among VI, SCC, and PFI in a nationally representative sample of American elder adults using data from the National Health and Nutrition Examination Survey (NHANES).

## Methods

### Study design and population

For this cross-sectional secondary analysis study, we included subjects aged 60 or older who participated in the 2005–2008 NHANES. The NHANES database is publicly available, and accessing requires no extra ethical or other approvals.

The NHANES is an ongoing survey aimed at evaluating the health and nutrition status of the U.S. civilian population. Annually, about 5,000 participants are recruited through a complex multistage probability sampling procedure. This sampling intentionally oversamples specific sub-populations, ensuring an adequate representation on a national scale. The survey consists of interviews and physical examinations, administered by well-trained research personnel. Rigorous adherence to standardized protocols guarantees the reliability of the collected data. More details regarding the design and procedures of the NHANES are available on the Centre for Disease Control and Prevention website (https://www.cdc.gov/nchs/nhanes).

According to the survey design of NHANES, the ultimate national estimates are then calculated by applying sample weight parameters. Weights can be viewed as measures of how many people in a target population are represented by a particular sampled participant, which is one of the characteristics of the study design for NHANES.

### Inclusion and exclusion criteria

**Fig. 1:**
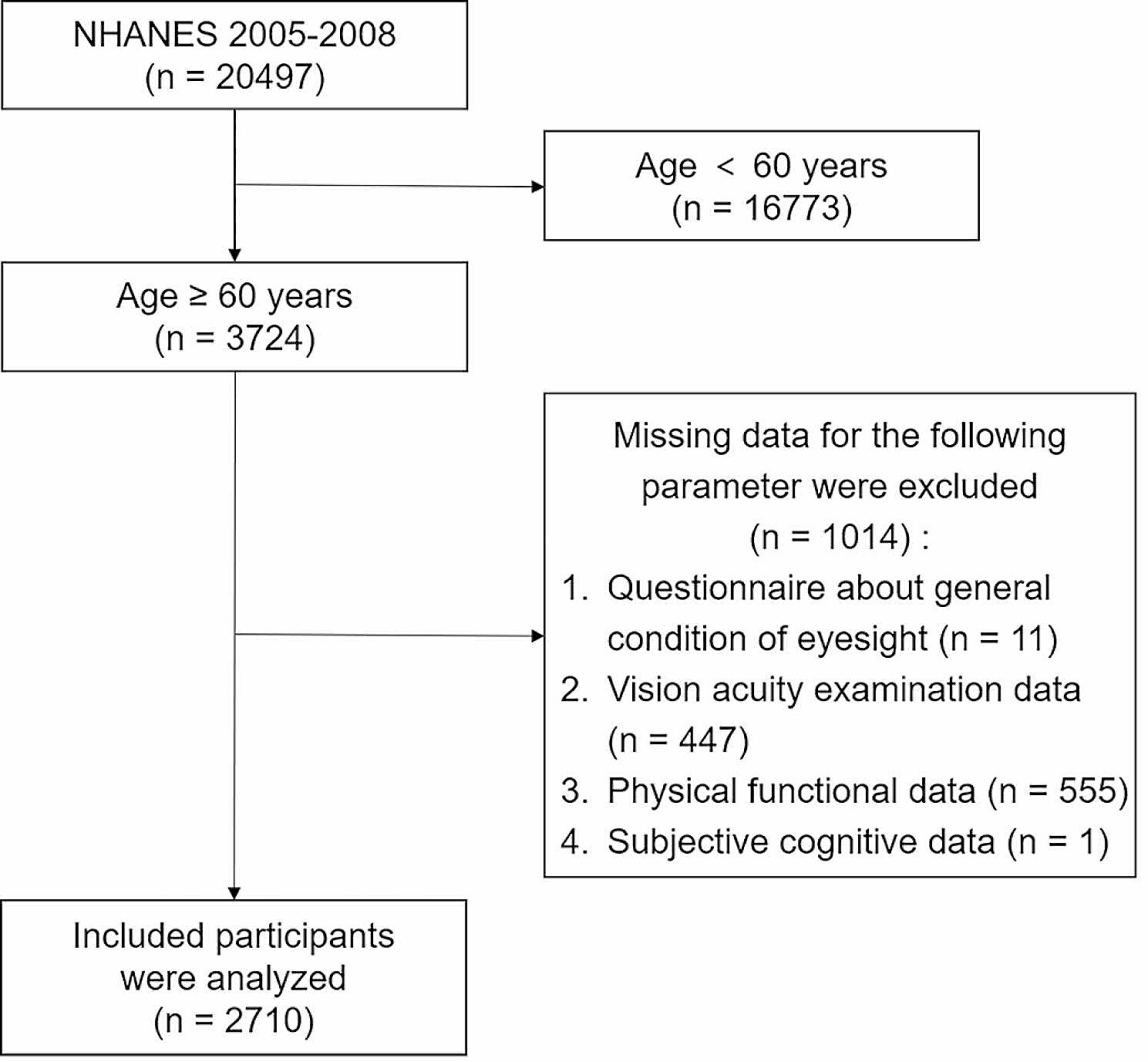
Flow chart of study subjects’ selection

This study was conducted on the older adult population aged ≥ 60 years. The screening criteria comprised 4 aspects, including VI assessment, SCC assessment, PFI assessment, and measurement of covariates. The specific procedure is detailed as follows (Fig. 1).

#### Visual impairment assessment

In the study, vision assessment involved both subjective self-reported eyesight conditions and objective visual acuity (VA) tests. Subjective visual impairment (SVI) was established based on interview responses. Participants were directly questioned about the general condition of eyesight (‘At the present time, would you say your eyesight, with glasses or contact lenses if you wear them, is…’). They were categorized as non-SVI if their answers were ‘excellent, or ‘good’. While, they were categorized as SVI if their answers were ‘fair’, ‘poor’, or ‘very poor’. Any missing data coded as ‘Don’t know’ ‘Refused’ or ‘Missing’, were excluded. Objective visual impairment (OVI) was determined by VA test results with whatever correction the participant usually wears, if any. More details about vision assessment can be found elsewhere [19]. Participants with a presenting VA of 20/40 or better in either eye were classified as non-OVI. Those with presenting VA worse than 20/40 in the better eye were classified as OVI. Participants lacking data for presenting VA in both eyes were excluded from the analysis.

#### Subjective cognitive complaints assessment

The definition of SCC refers to the previous study [17] by asking, ‘Are you limited in any way because of difficulty remembering or because you experience periods of confusion?’ The participant was classified as SCC with a ‘yes’ response. All missing data defined the same as SVI were excluded.

#### Physical functional impairment assessment

PFI was assessed via 20 interview items, subcategorized into five primary domains (Table 1): (1) activities of daily living (ADL), (2) instrumental activities of daily living (IADL), (3) leisure and social activities (LSA), (4) lower-extremity mobility (LEM), and (5) general physical activities (GPA) [20–22]. For each item, participants were defined as having difficulty if the answer was ‘some difficulty’, ‘much difficulty’, or ‘unable to do’. The domain was classified as having difficulty if one or more items were judged as having difficulty. If three or more of the five major domains were judged as having difficulty, participants were classified as PFI. Similarly, all missing data, defined as before, were excluded.

**Table 1:** Classification of interview items for physical functional impairment assessment

Classification	Interview items
	‘By yourself and without using any special equipment, how much difficulty do you have...
ADL	• Getting in and out of bed • Eating • Dressing yourself
IADL	• Managing money • Doing house chores • Preparing meals
LSA	• Going to the movies • Attending social events • Performing leisure activity at home
LEM	• Walking a quarter mile • Walking up to 10 steps • Walking between rooms on the same floor • Stooping/kneeling/crouching • Standing up from an armless chair
GPA	• Lifting or carrying heavy objects • Standing for long periods • Sitting for long periods • Reaching up overhead • Grasping/holding small objects • Moving large objects

ADL, activities of daily living; IADL, instrumental activities of daily living; LSA, leisure and social activities; LEM, lower-extremity mobility; GPA, general physical activities

#### Measurement of covariates

Based on previous studies [23–25], several potential covariates, including age, gender, race/ ethnicity, education level, marital status, the number of household members, the ratio of family income to poverty, smoking status, alcohol intake, body mass index (BMI), hypertension, diabetes, self-reported health status, and depression, were considered. The ratio of family income to poverty is used to measure the general economic status of the household and is categorized into < 1 (below the poverty level) and ≥ 1 (above the poverty level). For smoking status, the participants were asked to report whether they ever smoked > 100 cigarettes and whether they now smoke cigarettes. According to their answers, the participants were categorized into: never, former, or current smokers. According to the self-reported questionnaire, the alcohol intake was calculated and categorized into four groups based on the number and frequency of alcoholic beverages: none, 1–5 drinks/month, 5–10 drinks/month, and 10 or more drinks/month. A drink meant a 12 oz. beer, a 5 oz. of wine or one and a half ounces of liquor. BMI was divided into four groups: underweight (< 18.5 kg/m^2^), normal weight (18.5–25.0 kg/m^2^), overweight (25.0–30.0 kg/m^2^), and obese (≥ 30 kg/m^2^). Hypertension was defined by the answer to the question: ‘Have you ever been told by a doctor or other health professional that you had hypertension, also called high blood pressure?’ and was judged to those answering ‘yes’. Diabetes was identified as it met one of the following criteria: (a) hemoglobin A1C concentration (HbA1c) ≥ 6.5%, or fasting plasma glucose ≥ 7.0 mmol/L; (b) those who answered ‘yes’ in the following questions: ‘have you ever been told by a doctor that you have diabetes or sugar diabetes?’ or ‘Are you taking insulin now?’. Data on self-reported general health were obtained from the questionnaire and were divided into two categories (Good/excellent vs. Poor/fair). Depression was judged based on the Patient Health Questionnaire (PHQ-9), a quantification tool for evaluating the frequency of depressive symptoms in the past two weeks [26]. The total score of PHQ-9 was 27 points, and the cut-off for depression was a score ≥ 10 points, according to previous studies [27]. If the covariates of enrolled subjects were missing, we will show them in the result directly. The missing data were not included in the statistical analysis.

### Statistical analysis

Given that NHANES employed a complex, multistage probability sampling design, the weighting procedures were used to ensure that it was nationally representative of the U.S. population. The analysis was conducted using both original data and weighted estimates, as per the NHANES analytic guidelines (https://wwwn.cdc.gov/nchs/nhanes/analyticguidelines.aspx). Weighted values for each cycle can be obtained directly from the database. Weights for combined cycles were calculated according to guidelines. The characteristics of SVI and OVI were summarized using descriptive analyses. Categorical variables were described as numbers (n) with percentages (%) and analyzed by chi-square tests. All continuous data was non-normal distributed, and tested for normality with the Shapiro-Wilk test. Non-normal continuous variables were described as median (M) with interquartile (Q1, Q3) and analyzed by Wilcoxon signed-rank test. Univariate analyses were used to evaluate the effects of covariables on VI, SCC, or PFI. The binary logistic regression models were applied to test the crude and adjusted pairwise association among VI, SCC, and PFI. The crude model was unadjusted and only estimated the association between the two of them. The adjusted model was adjusted for confounding factors, which were selected according to the univariate analysis results and the findings of existing literature. We constructed an additive interaction model to test the presence of the interaction of two variables. That is, the model was used to estimate whether the joint effect of VI and SCC was greater than the sum of the two independent effects. For additive interaction, relative excess risk due to interaction (RERI), attributable proportion due to interaction (AP), and synergy index (S) were applied to assess. RERI > 0, AP > 0, or S > 1 meant positive interaction; RERI < 0, AP < 0, or S < 1 meant negative interaction. There was no biological interaction when the 95%CI of RERI or AP contained 0 or the 95%CI of S contained 1. All data were collated and analyzed with *R* version 3.0.1 (*R* Core Team, *R* Foundation for Statistical Computing, Vienna, Austria) and *RStudio* (*RStudio* Inc., Boston, MA, US). *P* value < 0.05 was considered a statistically significant difference.

## Results

A total of 2,710 subjects (weighted *n* = 38,966,687) who were 60 years or older were included. According to the SVI criteria, 613 (22.62%) were diagnosed as having SVI (48.61% in females, median age: 69 (63, 78)). Demographic and health-related characteristics for the included subjects (or weighted sample estimates) classified by SVI are shown in Table 2 and supplementary Table 1. Compared with subjects without SVI, those with SVI were more likely to be older, ethnic minority, less than a college graduate, unmarried, living in multi-person households, under the poverty level, a smoker, drinker, diabetes, poor self-reported health, and depression. The prevalence of CSS and PFI in the SVI group was 16.15% (weighted prevalence: 16.93%) and 35.07% (weighted prevalence: 36.88%); those were all significantly higher than without SVI. According to OVI criteria, 295 (10.89%) were OVI, of whom 53.22% were female. The distribution of age, education level, marital status, total number of people in the household, family income to poverty ratio, BMI, and self-reported general health for included subjects in the OVI group differed from those in the non-OVI group, and the differences were statistical significance. Their demographic and health-related characteristics (original data and weighted estimates) are detailed in Table 3 and supplementary Table 2. Similarly, the prevalence of CSS and PFI for OVI also was higher than non-SVI significantly (unweighted prevalence of SCC: 13.90%, weighted prevalence of SCC: 14.03%, unweighted prevalence of PFI: 33.22%, weighted prevalence of PFI: 33.81%).

**Table 2:** Demographic and health-related characteristics of study subjects with and without subjective visual impairment

Characteristic	Subjects without subjective visual impairment, *n* = 2097	Subjects with subjective visual impairment, *n* = 613	*P* ^#^
Gender, n (%)			0.861
Female	1,011 (48.21%)	298 (48.61%)	
Male	1,086 (51.79%)	315 (51.39%)	
Age, M (Q1, Q3)	70 (64, 76)	69 (63, 78)	0.712
Age, n (%)			**0.002**
60–69 years	1,040 (49.59%)	315 (51.39%)	
70–79 years	729 (34.76%)	174 (28.38%)	
80 + years	328 (15.64%)	124 (20.23%)	
Race/ethnicity, n (%)			**< 0.001**
Mexican American	236 (11.25%)	127 (20.72%)	
Other Hispanic	125 (5.96%)	58 (9.46%)	
Non-Hispanic White	1,300 (61.99%)	271 (44.21%)	
Non-Hispanic Black	377 (17.98%)	148 (24.14%)	
Other/multiracial	59 (2.81%)	9 (1.47%)	
Education level, n (%)			**< 0.001**
< College graduate	1,651 (78.73%)	545 (88.91%)	
≥ College graduate	446 (21.27%)	68 (11.09%)	
Marital status, n (%)			**< 0.001**
Married/living with partner	1,341 (63.95%)	326 (53.18%)	
Widowed/separated/divorced	684 (32.62%)	251 (40.95%)	
Never married	72 (3.43%)	36 (5.87%)	
Total number of people in the Household, n (%)			**< 0.001**
1	478 (22.79%)	144 (23.49%)	
2–5	1,489 (71.01%)	382 (62.32%)	
≥ 5	130 (6.20%)	87 (14.19%)	
Ratio of family income to poverty, M (Q1, Q3)	2.45 (1.46, 4.38)	1.65 (0.98, 2.71)	**< 0.001**
Ratio of family income to poverty, n (%)			**< 0.001**
< 1	214 (11.05%)	142 (25.49%)	
≥ 1	1,723 (88.95%)	415 (74.51%)	
Missing	160	56	
Smoking status, n (%)			**< 0.001**
Never	991 (47.26%)	270 (44.05%)	
Former	883 (42.11%)	233 (38.01%)	
Current	223 (10.63%)	110 (17.94%)	
Alcohol intake, n (%)			**0.006**
Non-drinker	784 (37.39%)	255 (41.60%)	
1–5 drinks/month	857 (40.87%)	264 (43.07%)	
5–10 drinks/month	103 (4.91%)	23 (3.75%)	
10 + drinks/month	353 (16.83%)	71 (11.58%)	
Body mass index (BMI, kg/m2), n (%)			0.428
Underweight	26 (1.25%)	10 (1.65%)	
Normal weight	563 (27.07%)	155 (25.62%)	
Overweight	799 (38.41%)	220 (36.36%)	
Obesity	692 (33.27%)	220 (36.36%)	
Missing	17	8	
Hypertension, n (%)			0.074
Yes	1,153 (54.98%)	362 (59.05%)	
No	944 (45.02%)	251 (40.95%)	
Diabetes, n (%)			**< 0.001**
Yes	490 (23.37%)	201 (32.79%)	
No	1,607 (76.63%)	412 (67.21%)	
Self-reported general health, n (%)			**< 0.001**
Good/excellent	1,646 (81.85%)	313 (53.32%)	
Poor/fair	365 (18.15%)	274 (46.68%)	
Missing	86	26	
Depression, n (%)			**< 0.001**
No	1,944 (97.25%)	530 (91.22%)	
Yes	55 (2.75%)	51 (8.78%)	
Missing	98	32	
Subjective cognitive complaints, n (%)			**< 0.001**
No	1,947 (92.85%)	514 (83.85%)	
Yes	150 (7.15%)	99 (16.15%)	
Physical functional impairment, n (%)			**< 0.001**
No	1,743 (83.12%)	398 (64.93%)	
Yes	354 (16.88%)	215 (35.07%)	
Activities of daily living, n (%)			**< 0.001**
No	1,886 (89.94%)	475 (77.49%)	
Yes	211 (10.06%)	138 (22.51%)	
Instrumental activities of daily living, n (%)			**< 0.001**
No	1,802 (85.93%)	448 (73.08%)	
Yes	295 (14.07%)	165 (26.92%)	
Leisure and social activities, n (%)			**< 0.001**
No	1,928 (91.94%)	485 (79.12%)	
Yes	169 (8.06%)	128 (20.88%)	
Lower-extremity mobility, n (%)			**< 0.001**
No	1,173 (55.94%)	250 (40.78%)	
Yes	924 (44.06%)	363 (59.22%)	
General physical activities, n (%)			**< 0.001**
No	1,157 (55.17%)	254 (41.44%)	
Yes	940 (44.83%)	359 (58.56%)	

^#^ Pearson’s Chi-squared test; Wilcoxon rank sum test; Bold *P* values signal statistical significance

**Table 3:** Demographic and health-related characteristics of study subjects with and without objective visual impairment

Characteristic	Subjects without objective visual impairment, *n* = 2415	Subjects with objective visual impairment, *n* = 295	*P* ^#^
Gender, n (%)			0.073
Female	1,152 (47.70%)	157 (53.22%)	
Male	1,263 (52.30%)	138 (46.78%)	
Age, M (Q1, Q3)	69 (63, 76)	74 (67, 80)	**< 0.001**
Age, n (%)			**< 0.001**
60–69 years	1,251 (51.80%)	104 (35.25%)	
70–79 years	812 (33.62%)	91 (30.85%)	
80 + years	352 (14.58%)	100 (33.90%)	
Race/ethnicity, n (%)			0.083
Mexican American	308 (12.75%)	55 (18.64%)	
Other Hispanic	163 (6.75%)	20 (6.78%)	
Non-Hispanic White	1,414 (58.55%)	157 (53.22%)	
Non-Hispanic Black	470 (19.46%)	55 (18.64%)	
Other/multiracial	60 (2.48%)	8 (2.71%)	
Education level, n (%)			**< 0.001**
< College graduate	1,934 (80.08%)	262 (88.81%)	
≥ College graduate	481 (19.92%)	33 (11.19%)	
Marital status, n (%)			**< 0.001**
Married/living with partner	1,534 (63.52%)	133s (45.08%)	
Widowed/separated/divorced	788 (32.63%)	147 (49.83%)	
Never married	93 (3.85%)	15 (5.08%)	
Total number of people in the Household, n (%)			**0.003**
1	542 (22.44%)	80 (27.12%)	
2–5	1,691 (70.02%)	180 (61.02%)	
≥ 5	182 (7.54%)	35 (11.86%)	
Ratio of family income to poverty, M (Q1, Q3)	2.29 (1.41, 4.09)	1.64 (1.00, 2.82)	**< 0.001**
Ratio of family income to poverty, n (%)			**< 0.001**
< 1	292 (13.09%)	64 (24.24%)	
≥ 1	1,938 (86.91%)	200 (75.76%)	
Missing	185	31	
Smoking status, n (%)			0.290
Never	1,113 (46.09%)	148 (50.17%)	
Former	1,007 (41.70%)	109 (36.95%)	
Current	295 (12.22%)	38 (12.88%)	
Alcohol intake, n (%)			0.067
Non-drinker	906 (37.52%)	133 (45.08%)	
1–5 drinks/month	1,007 (41.70%)	114 (38.64%)	
5–10 drinks/month	115 (4.76%)	11 (3.73%)	
10 + drinks/month	387 (16.02%)	37 (12.54%)	
Body mass index (BMI, kg/m2), n (%)			**0.029**
underweight	32 (1.34%)	4 (1.39%)	
normal weight	622 (25.95%)	96 (33.33%)	
overweight	911 (38.01%)	108 (37.50%)	
obesity	832 (34.71%)	80 (27.78%)	
Missing	18	7	
Hypertension, n (%)			0.528
Yes	1,345 (55.69%)	170 (57.63%)	
No	1,070 (44.31%)	125 (42.37%)	
Diabetes, n (%)			0.051
Yes	602 (24.93%)	89 (30.17%)	
No	1,813 (75.07%)	206 (69.83%)	
Self-reported general health, n (%)			**< 0.001**
Good/excellent	1,777 (76.56%)	182 (65.70%)	
Poor/fair	544 (23.44%)	95 (34.30%)	
Missing	94	18	
Depression, n (%)			0.790
No	2,214 (95.93%)	260 (95.59%)	
Yes	94 (4.07%)	12 (4.41%)	
Missing	107	23	
Subjective cognitive complaints, n (%)			**0.003**
No	2,207 (91.39%)	254 (86.10%)	
Yes	208 (8.61%)	41 (13.90%)	
Physical functional impairment, n (%)			**< 0.001**
No	1,944 (80.50%)	197 (66.78%)	
Yes	471 (19.50%)	98 (33.22%)	
Activities of daily living, n (%)			**0.003**
No	2,120 (87.78%)	241 (81.69%)	
Yes	295 (12.22%)	54 (18.31%)	
Instrumental activities of daily living, n (%)			**< 0.001**
No	2,030.00 (84.06%)	220.00 (74.58%)	
Yes	385.00 (15.94%)	75.00 (25.42%)	
Leisure and social activities, n (%)			**< 0.001**
No	2,174.00 (90.02%)	239.00 (81.02%)	
Yes	241.00 (9.98%)	56.00 (18.98%)	
Lower-extremity mobility, n (%)			**0.010**
No	1,289 (53.37%)	134 (45.42%)	
Yes	1,126 (46.63%)	161 (54.58%)	
General physical activities, n (%)			**0.022**
No	1,276 (52.84%)	135 (45.76%)	
Yes	1,139 (47.16%)	160 (54.24%)	

^#^ Pearson’s Chi-squared test; Wilcoxon rank sum test; Bold *P* values signal statistical significance

The associations between VI and PFI are shown in Table 4. For PFI, SVI had a significant positive association with them [weighted OR (95%CI): 3.11 (2.25, 4.31), *P* < 0.001]. After adjustment for age, gender, education level, marital status, the ratio of family income to poverty, alcohol intake, hypertension, diabetes, self-reported general health, and depression, those with SVI remained at a higher risk of PFI [weighted OR (95%CI): 1.90 (1.32, 2.72), *P* = 0.002]. Similarly, compared to those without OVI, those with OVI were positively associated with the risk of PFI in the crude model [weighted OR (95%CI): 2.35 (1.53, 3.61), *P* < 0.001]. When adjusting for possible confounding variables further, OVI was statistically associated with PFI [weighted OR (95%CI): 1.84 (1.07, 3.17), *P* = 0.030].

**Table 4:** Association of physical functional impairment between visual impairment and subjective cognitive complaints

Characteristic		Unweighted			Weighted		
		OR	(95% CI)	*P*	OR	(95% CI)	*P*
Subjective visual impairment	Crude	2.66	(2.17, 3.25)	**< 0.001**	3.11	(2.25, 4.31)	**< 0.001**
	Adjusted	1.77	(1.39, 2.25)	**< 0.001**	1.90	(1.32, 2.72)	**0.002**
Objective visual impairment	Crude	2.05	(1.57, 2.66)	**< 0.001**	2.35	(1.53, 3.61)	**< 0.001**
	Adjusted	1.60	(1.16, 2.18)	**0.004**	1.84	(1.07, 3.17)	**0.030**
Subjective cognitive complaints	Crude	5.33	(4.07, 6.98)	**< 0.001**	5.02	(3.40, 7.40)	**< 0.001**
	Adjusted	3.87	(2.82, 5.33)	**< 0.001**	3.29	(2.01, 5.38)	**< 0.001**

OR = Odds Ratio, CI = Confidesnce Interval; Bold *P* values signal statistical significance

In addition, there was a statistically significant association between SCC and PFI in the crude model (Table 4) [weighted OR (95%CI): 5.02 (3.40, 7.40), *P* < 0.001]. After adjustment, a statistical association between SCC and PFI was found as well [weighted OR (95%CI): 3.29 (2.01, 5.38), *P* < 0.001].

As Table 5 listed, there was an additive interaction between SVI and SCC on PFI before and after weighting [weighted RERI (95%CI): 6.52 (0.60, 16.52), weighted AP (95%CI): 0.52 (0.04, 0.72), weighted SI (95%CI): 2.30 (1.11, 4.75)]. Among them, the AP value of 0.52 meant that 52% of PFI cases were caused by the interaction between SVI and SCC in this study. However, there was no interaction between OVI and SCC on PFI [weighted RERI (95%CI): 6.33 (-1.07, 26.07), weighted AP (95%CI): 0.52 (-0.34, 0.74), weighted SI (95%CI): 2.30 (0.81, 6.52)].

**Table 5:** Interactive effect analysis of visual impairments and subjective cognitive complaints

Measures	Unweighted		Weighted	
	Estimates	95% CI	Estimates	95% CI
Subjective visual impairments and subjective cognitive complaints				
RERI	8.38	**(3.29, 16.13)**	6.52	**(0.60, 16.52)**
AP	0.61	**(0.34, 0.75)**	0.52	**(0.04, 0.72)**
SI	2.92	**(1.65, 5.16)**	2.30	**(1.11, 4.75)**
Objective visual impairments and subjective cognitive complaints				
RERI	3.53	(-1.41, 12.54)	6.33	(-1.07, 26.07)
AP	0.36	(-0.32, 0.64)	0.52	(-0.34, 0.74)
SI	1.68	(0.77, 3.65)	2.30	(0.81, 6.52)

RERI, relative excess risk due to interaction; AP, attributable proportion of interaction; S, synergy index; CI, confidence interval; Bold *P* values signal statistical significance

## Discussion

In this study, we focused on three prevalent health indicators: vision, cognitive function, and physical activity, all of which are relevant to the well-being of older adults. Utilizing the data from a large nationally representative sample of the older U.S. population, we observed that SVI, OVI, and SCC had associations with PFI among older adults. Furthermore, there was a synergistic interaction between SVI and SCC on PFI. The results suggested that improving VI and cognitive impairment of elder adults may be beneficial to the prevention of PFI.

A study reported that among aged 40 and older people in the US in 2015, there were 3.22 million people with visual impairment, and 1.02 million people were blind. It has grown by more than 20% in the past decade and is expected to double by 2050 [28]. The increasing prevalence of VI in the ageing population places a significant burden on society and healthcare systems. As an essential sensorium, vision function is always involved in physical and mental activity. The visual system is nonnegligible in recognizing the surroundings and processing, planning, and organizing actions. Previous studies have reported the important role of vision in physical and mental activities [29]. Our study complements additional evidence by observing a significant positive association between VI and PFI in American older adults at a national level. It is known that physical activity is an indicator of overall health and fitness. Due to reduced or lack of visual perception, people with visual impairments may have reduced balance and increased fall-related risks [30]. In turn, concerns about the risks associated with exercise may further increase the degree of physical activity restriction [31, 32]. A previous study has also shown that people with worse vision had lower intensity or frequency of physical activity [33]. Cai et al. found that for people with worse visual acuity, contrast sensitivity, and visual fields, their daily activities became less, shorter, and more fragmented [18].

The association between VI and PFI in older adults is evident, although the direction of causality is still not fully understood. Perhaps VI could be the cause of PFI, or it could be located downstream of PFI, or that causality could be bidirectional. An Australian longitudinal cohort study found that cataract surgery on the first and second eyes independently increased the amount of time spent in moderate leisure physical activity [34]. This suggests that interventions targeting VI, such as cataract surgery, may have positive effects on physical activity levels. Removing VI and promoting physical activity in older adults may have significant implications for their overall health and quality of life. On the other hand, a population-based prospective cohort study found that high levels of physical activity, especially long-term physical activity, may be associated with a reduced risk of age-related cataracts. Conversely, low activity levels may be associated with an increased risk of cataracts [35]. These findings suggest a potential reciprocal relationship between the degree of physical activity and the development of VI. Therefore, subsequent studies will focus on more specific relationships and mechanisms based on high-quality evidence.

Moreover, the prevalence of recognize-related disorders increases with population ageing, and SCC has been noted gradually and recognized as more than just an accompanying symptom of ageing. A population-based clinical trial indicated that SCC served as a vital sign of subjective cognitive decline, a pre-clinical stage of Alzheimer’s disease [36]. A meta-analysis of prospective longitudinal studies revealed that individuals with self-reported SCC have 1.27-fold (95%CI: 1.06–1.53, *P* < 0.001) higher risks of transition to mild cognitive impairment and/or dementia [37]. SCC could exist as an independent disorder or coexist with physical or mental disorders. Marrero-Polegre et al. assessed 30 healthy older adults from the community and found that lower visual processing speed was associated with more severe SCC [38]. Zhu Z et al. found significant associations between age-related macular degeneration severity and SCC using NHANES data from 2005 to 2008 [17]. As AMD progresses, visual impairment develops gradually. A cross-sectional study based on data from the 2019 National Health Interview Survey demonstrated that sensory impairments, including visual or hearing impairments or both, were associated with an increased prevalence of SCC in older adults [39]. Another study of NHANES found walking impairment was associated with visual impairment, depression, and cognitive impairment, as well as with the number of impairment indicators [16]. Our study, which utilized a nationally representative sample of older adults, identified an association between PFI and SCC. The existing literature supported that regular physical activity could significantly reduce the risk of dementia and Alzheimer’s disease, although the optimal intensity and type of activity remained controversial [40]. The effect of physical activity on cognitive function may be attributed to various mechanisms. Previous studies have found that physical activity contributes to increasing brain volume and reduced brain atrophy, particularly in the hippocampus [41, 42]. Other researchers considered that the positive influence of physical activity on cognitive function may be mediated through increased blood flow and perfusion in the brain, leading to enhanced neurogenesis [43, 44].

The present study differed from previous studies by exploring the additive interaction among SVI, PFI, and SCC, in addition to their association with each other. Clinicians should adopt a comprehensive approach that addresses both existing dysfunctions and aims to prevent and manage other potential dysfunctions. The interaction analysis assesses whether the effects of one risk factor differ across levels of another. From the public health point of view, it is more relevant to assess interactions on additive models [45, 46]. Specific to this study, it suggested that the joint impact of SVI and SCC may magnify in individuals with PFI. It implied that improving vision or cognitive function may provide additive benefits for individuals with PFI. The mechanisms underlying the relationship between VI/SCC and PFI are still uncertain, and these mechanisms deserve further investigation. It is worthwhile to recognize that statistical additive interactions in the model do not necessarily imply a causal interactive relationship [45]. Further demonstration of this definitive relationship remains in longitudinal studies conducted on specific population groups.

This study has two significant strengths. Firstly, we utilized large-scale, high-quality, representative data from the NHANES on older Americans population. Secondly, while previous studies have shown that VI, SCC, and PFI were prevalent disabilities in older adults and had pairwise associations between variables, scarcely any studies have investigated the interaction among the three. In this study, we found that there was a significant positive interaction between SVI and SCC on FPI among the elderly, indicating the significance of jointed effects between SVI and SCC for physical activity, and overall health in older adults. Nevertheless, there are certain limitations in the following aspects. Firstly, the cross-sectional study designs based on NHANES data were restricted to draw causal inferences. Future researches need to improve the study design and investigate the additional role of improved vision in slowing down or preventing the progression of other disabilities in older adults with PFI/SCC. Secondly, the data on physical activity relied on self-reported questionnaires, which may be susceptible to recall bias and differ from those obtained through objective testing. Thirdly, the assessment of SCC was based on a single question, which could be more susceptible to interference. However, it is worth noting that the single-item measure of SCC has been approved by the Subjective Cognitive Decline Initiative Working Group in their published white paper, and many studies have already utilized this measure of SCC [36]. Finally, it’s important to note that the vision examinations were conducted over a specific time frame, specifically speaking in the cycles of 2005–2008. Until now, previous articles that needed data related to vision were from these cycles. This time gap between then and the present should be considered when interpreting the results.

## Conclusion

In conclusion, these findings observed that VI and SCC were significantly associated with PFI in older adults. Especially, there was a significant synergistic interaction between SVI and SCC, amplifying their joint impact on PFI. More attention should be paid to identifying and mitigating the potential risk factors affecting elderly health and their interaction effects.

### Electronic supplementary material

Below is the link to the electronic supplementary material.

Supplementary Material 1

Supplementary Material 2

## Data Availability

All data relevant to the study is publicly available from the NHANES website (https://wwwn.cdc.gov/nchs/nhanes/Default.aspx).
