# Supplementary material for: Interaction between visual impairment and subjective cognitive complaints on physical activity impairment in U.S. older adults: NHANES 2005–2008
Source: BMC Geriatr. 2024 Feb 17;24:167. doi: 10.1186/s12877-024-04739-2 (PMC10874547; doi:10.1186/s12877-024-04739-2)
Supplement: Supplementary file 1 — Supplementary Material 1 [file 12877_2024_4739_MOESM1_ESM.docx]

Supplementary table 1. Demographic and health-related characteristics of study subjects with and without subjective visual impairment using weighted and complex sampling design of the National Health and Nutrition Examination Survey

| Characteristic | Subjects without subjective visual impairment,  n = 32548909 | Subjects with subjective visual impairment,  n = 6417778 | *P ^#^* |
| --- | --- | --- | --- |
| Gender, n (%) |  |  | 0.957 |
| Female | 17,544,829 (53.90%) | 3,449,769 (53.75%) |  |
| Male | 15,004,080 (46.10%) | 2,968,009 (46.25%) |  |
| Age, M (Q1, Q3) | 68 (64, 75) | 70 (64, 79) | **0.007** |
| Age, n (%) |  |  | **<0.001** |
| 60-69 years | 17,554,832 (53.93%) | 3,124,287 (48.68%) |  |
| 70-79 years | 10,896,499 (33.48%) | 1,801,745 (28.07%) |  |
| 80+ years | 4,097,578 (12.59%) | 1,491,746 (23.24%) |  |
| Race/ethnicity, n (%) |  |  | **<0.001** |
| Mexican American | 948,288 (2.91%) | 511,427 (7.97%) |  |
| Other Hispanic | 677,353 (2.08%) | 299,975 (4.67%) |  |
| Non-Hispanic White | 27,550,657 (84.64%) | 4,628,698 (72.12%) |  |
| Non-Hispanic Black | 2,118,791 (6.51%) | 857,304 (13.36%) |  |
| Other/multiracial | 1,253,820 (3.85%) | 120,374 (1.88%) |  |
| Education level, n (%) |  |  | **0.012** |
| < College graduate | 23,877,506 (73.36%) | 5,249,262 (81.79%) |  |
| ≥ College graduate | 8,671,403 (26.64%) | 1,168,516 (18.21%) |  |
| Marital status, n (%) |  |  | **<0.001** |
| Married/living with partner | 22,030,612 (67.68%) | 3,607,490 (56.21%) |  |
| Widowed/separated/divorced | 9,714,561 (29.85%) | 2,479,601 (38.64%) |  |
| Never married | 803,736 (2.47%) | 330,687 (5.15%) |  |
| Total number of people in the Household, n (%) |  |  | **<0.001** |
| 1 | 7,415,572 (22.78%) | 1,697,539 (26.45%) |  |
| 2-5 | 2,4231,822 (74.45%) | 4,242,258 (66.10%) |  |
| ≥5 | 901,515 (2.77%) | 477,981 (7.45%) |  |
| Ratio of family income to poverty, M (Q1, Q3) | 2.92 (1.83, 4.92) | 1.95 (1.22, 3.51) | **<0.001** |
| Ratio of family income to poverty, n (%) |  |  | **<0.001** |
| < 1 | 1,789,687 (5.92%) | 1,014,208 (17.50%) |  |
| ≥ 1 | 28,441,367 (94.08%) | 4,781,515 (82.50%) |  |
| Unknown | 2,317,854 | 622,056 |  |
| Smoking status, n (%) |  |  | **0.010** |
| Never | 15,036,250 (46.20%) | 2,780,190 (43.32%) |  |
| Former | 14,038,161 (43.13%) | 2,581,263 (40.22%) |  |
| Current | 3,474,498 (10.67%) | 1,056,325 (16.46%) |  |
| Alcohol intake, n (%) |  |  | **0.032** |
| Non-drinker | 11,363,982 (34.91%) | 2,559,172 (39.88%) |  |
| 1-5 drinks/month | 12,816,402 (39.38%) | 2,643,951 (41.20%) |  |
| 5-10 drinks/month | 1,859,136 (5.71%) | 293,875 (4.58%) |  |
| 10+ drinks/month | 6,509,389 (20.00%) | 920,780 (14.35%) |  |
| Body mass index (BMI, kg/m2), n (%) |  |  | 0.735 |
| Underweight | 382,056 (1.18%) | 116,129 (1.83%) |  |
| Normal weight | 9,065,327 (28.02%) | 1,658,421 (26.10%) |  |
| Overweight | 12,600,394 (38.94%) | 2,448,670 (38.53%) |  |
| Obesity | 10,309,561 (31.86%) | 2,131,548 (33.54%) |  |
| Unknown | 191,571 | 63,011 |  |
| Hypertension, n (%) |  |  | 0.056 |
| Yes | 17,688,479 (54.34%) | 3,937,694 (61.36%) |  |
| No | 14,860,429 (45.66%) | 2,480,084 (38.64%) |  |
| Diabetes, n (%) |  |  | **<0.001** |
| Yes | 6,201,441 (19.05%) | 1,813,484 (28.26%) |  |
| No | 26,347,468 (80.95%) | 4,604,294 (71.74%) |  |
| Self-reported general health, n (%) |  |  | **<0.001** |
| Good/excellent | 27,198,449 (86.31%) | 3,893,395 (63.17%) |  |
| Poor/fair | 4,313,529 (13.69%) | 2,269,751 (36.83%) |  |
| Unknown | 1,036,931 | 254,632 |  |
| Depressive symptom, n (%) |  |  | **<0.001** |
| No | 30,491,877 (97.32%) | 5,541,365 (90.56%) |  |
| Yes | 839,438 (2.68%) | 577,850 (9.44%) |  |
| Unknown | 1,217,594 | 298,563 |  |
| Subjective cognitive complaints, n (%) |  |  | **<0.001** |
| No | 30,363,876 (93.29%) | 5,331,161 (83.07%) |  |
| Yes | 2,185,033 (6.71%) | 1,086,618 (16.93%) |  |
| Physical functional impairment, n (%) |  |  | **<0.001** |
| No | 27,404,913 (84.20%) | 4,050,629 (63.12%) |  |
| Yes | 5,143,996 (15.80%) | 2,367,150 (36.88%) |  |
| Activities of daily living, n (%) |  |  | **<0.001** |
| No | 29,673,130 (91.16%) | 5,010,598 (78.07%) |  |
| Yes | 2,875,779 (8.84%) | 1,407,180 (21.93%) |  |
| Instrumental activities of daily living, n (%) |  |  | **<0.001** |
| No | 28,198,550 (86.63%) | 4,556,735 (71.00%) |  |
| Yes | 4,350,359 (13.37%) | 1,861,043 (29.00%) |  |
| Leisure and social activities, n (%) |  |  | **<0.001** |
| No | 30,243,953 (92.92%) | 5,006,707 (78.01%) |  |
| Yes | 2,304,956 (7.08%) | 1,411,071 (21.99%) |  |
| Lower-extremity mobility |  |  | **<0.001** |
| No | 18,481,300 (56.78%) | 2,515,783 (39.20%) |  |
| Yes | 14,067,609 (43.22%) | 3,901,995 (60.80%) |  |
| General physical activities, n (%) |  |  | **<0.001** |
| No | 17,861,954 (54.88%) | 2,476,435 (38.59%) |  |
| Yes | 14,686,955 (45.12%) | 3,941,343 (61.41%) |  |

^#^ chi-squared test with Rao & Scott's second-order correction; Wilcoxon rank-sum test for complex survey samples
