# Supplementary material for: Interaction between visual impairment and subjective cognitive complaints on physical activity impairment in U.S. older adults: NHANES 2005–2008
Source: BMC Geriatr. 2024 Feb 17;24:167. doi: 10.1186/s12877-024-04739-2 (PMC10874547; doi:10.1186/s12877-024-04739-2)
Supplement: Supplementary file 2 — Supplementary Material 2 [file 12877_2024_4739_MOESM2_ESM.docx]

Supplementary table 2. Demographic and health-related characteristics of study subjects with and without objective visual impairment using weighted samples and complex sampling design of the National Health and Nutrition Examination Survey

| Characteristic | subjects without objective visual impairment,  n = 35487487 | subjects with objective visual impairment,  n = 3479200 | *P ^#^* |
| --- | --- | --- | --- |
| Gender, n (%) |  |  | 0.225 |
| Female | 18,955,103 (53.41%) | 2,039,496 (58.62%) |  |
| Male | 16,532,384 (46.59%) | 1,439,704 (41.38%) |  |
| Age, M (Q1, Q3) | 68 (63, 75) | 75 (67, 80) | **<0.001** |
| Age, n (%) |  |  | **<0.001** |
| 60-69 years | 19,485,134 (54.91%) | 1,193,985 (34.32%) |  |
| 70-79 years | 11,663,509 (32.87%) | 1,034,736 (29.74%) |  |
| 80+ years | 4,338,845 (12.23%) | 1,250,479 (35.94%) |  |
| Race/ethnicity, n (%) |  |  | 0.098 |
| Mexican American | 1,248,081 (3.52%) | 211,634 (6.08%) |  |
| Other Hispanic | 881,656 (2.48%) | 95,672 (2.75%) |  |
| Non-Hispanic White | 29,500,209 (83.13%) | 2,679,146 (77.00%) |  |
| Non-Hispanic Black | 2,630,817 (7.41%) | 345,278 (9.92%) |  |
| Other/multiracial | 1,226,724 (3.46%) | 147,469 (4.24%) |  |
| Education level, n (%) |  |  | **0.010** |
| < College graduate | 26,228,054 (73.91%) | 2,898,714 (83.32%) |  |
| ≥ College graduate | 9,259,434 (26.09%) | 580,485 (16.68%) |  |
| Marital status, n (%) |  |  | **<0.001** |
| Married/living with partner | 23,973,555 (67.55%) | 1,664,547 (47.84%) |  |
| Widowed/separated/divorced | 10,508,531 (29.62%) | 1,685,631 (48.45%) |  |
| Never married | 1,005,401 (2.83%) | 129,022 (3.71%) |  |
| Total number of people in the Household, n (%) |  |  | **0.010** |
| 1 | 8,099,672 (22.82%) | 1,013,439 (29.13%) |  |
| 2-5 | 26,222,549 (73.89%) | 2,251,530 (64.71%) |  |
| ≥5 | 1,165,266 (3.28%) | 214,231 (6.16%) |  |
| Ratio of family income to poverty, M (Q1, Q3) | 2.81 (1.75, 4.85) | 1.97 (1.21, 3.65) | **<0.001** |
| Ratio of family income to poverty, n (%) |  |  | **<0.001** |
| < 1 | 2,328,828 (7.08%) | 475,067 (15.21%) |  |
| ≥ 1 | 30,574,398 (92.92%) | 2,648,485 (84.79%) |  |
| Unknown | 2,584,261 | 355,648 |  |
| Smoking status, n (%) |  |  | 0.549 |
| Never | 16,106,214 (45.39%) | 1,710,226 (49.16%) |  |
| Former | 15,190,819 (42.81%) | 1,428,605 (41.06%) |  |
| Current | 4,190,453 (11.81%) | 340,369 (9.78%) |  |
| Alcohol intake, n (%) |  |  | **0.017** |
| Non-drinker | 12,371,114 (34.86%) | 1,552,040 (44.61%) |  |
| 1-5 drinks/month | 14,135,867 (39.83%) | 1,324,486 (38.07%) |  |
| 5-10 drinks/month | 2,015,377 (5.68%) | 137,634 (3.96%) |  |
| 10+ drinks/month | 6,965,129 (19.63%) | 465,040 (13.37%) |  |
| Body mass index (BMI, kg/m2), n (%) |  |  | 0.372 |
| underweight | 457,602 (1.30%) | 40,583 (1.19%) |  |
| normal weight | 9,650,798 (27.35%) | 1,072,950 (31.35%) |  |
| overweight | 13,646,815 (38.67%) | 1,402,249 (40.97%) |  |
| obesity | 11,534,233 (32.68%) | 906,876 (26.50%) |  |
| Unknown | 198,039 | 56,542 |  |
| Hypertension, n (%) |  |  | 0.231 |
| Yes | 19,562,527 (55.13%) | 2,063,646 (59.31%) |  |
| No | 15,924,960 (44.87%) | 1,415,554 (40.69%) |  |
| Diabetes, n (%) |  |  | 0.171 |
| Yes | 7,150,244 (20.15%) | 864,681 (24.85%) |  |
| No | 28,337,243 (79.85%) | 2,614,519 (75.15%) |  |
| Self-reported general health, n (%) |  |  | **<0.001** |
| Good/excellent | 28,640,718 (83.28%) | 2,451,125 (74.59%) |  |
| Poor/fair | 5,748,120 (16.72%) | 835,160 (25.41%) |  |
| Unknown | 1,098,648 | 192,915 |  |
| Depressive symptom, n (%) |  |  | 0.550 |
| No | 32,952,608 (96.29%) | 3080,634 (95.37%) |  |
| Yes | 1,267,889 (3.71%) | 149,399 (4.63%) |  |
| Unknown | 1,266,990 | 249,167 |  |
| Subjective cognitive complaints, n (%) |  |  | **0.007** |
| No | 32,703,898 (92.16%) | 2,991,138 (85.97%) |  |
| Yes | 2,783,589 (7.84%) | 488,061 (14.03%) |  |
| Physical functional impairment, n (%) |  |  | **<0.001** |
| No | 29,152,802 (82.15%) | 2,302,740 (66.19%) |  |
| Yes | 6,334,685 (17.85%) | 1,176,460 (33.81%) |  |
| Activities of daily living, n (%) |  |  | **0.017** |
| No | 31,769,558 (89.52%) | 2,914,170 (83.76%) |  |
| Yes | 3,717,929 (10.48%) | 565,030 (16.24%) |  |
| Instrumental activities of daily living, n (%) |  |  | **<0.001** |
| No | 30,212,524 (85.14%) | 2,542,761 (73.08%) |  |
| Yes | 5,274,963 (14.86%) | 936,439 (26.92%) |  |
| Leisure and social activities, n (%) |  |  | **<0.001** |
| No | 32,459,188 (91.47%) | 2,791,472 (80.23%) |  |
| Yes | 3,028,299 (8.53%) | 687,728 (19.77%) |  |
| Lower-extremity mobility, n (%) |  |  | **0.010** |
| No | 19,432,693 (54.76%) | 1,564,390 (44.96%) |  |
| Yes | 16,054,794 (45.24%) | 1,914,810 (55.04%) |  |
| General physical activities, n (%) |  |  | **0.036** |
| No | 18,771,155 (52.90%) | 1,567,234 (45.05%) |  |
| Yes | 16,716,332 (47.10%) | 1,911,966 (54.95%) |  |

^#^ chi-squared test with Rao & Scott's second-order correction; Wilcoxon rank-sum test for complex survey samples
